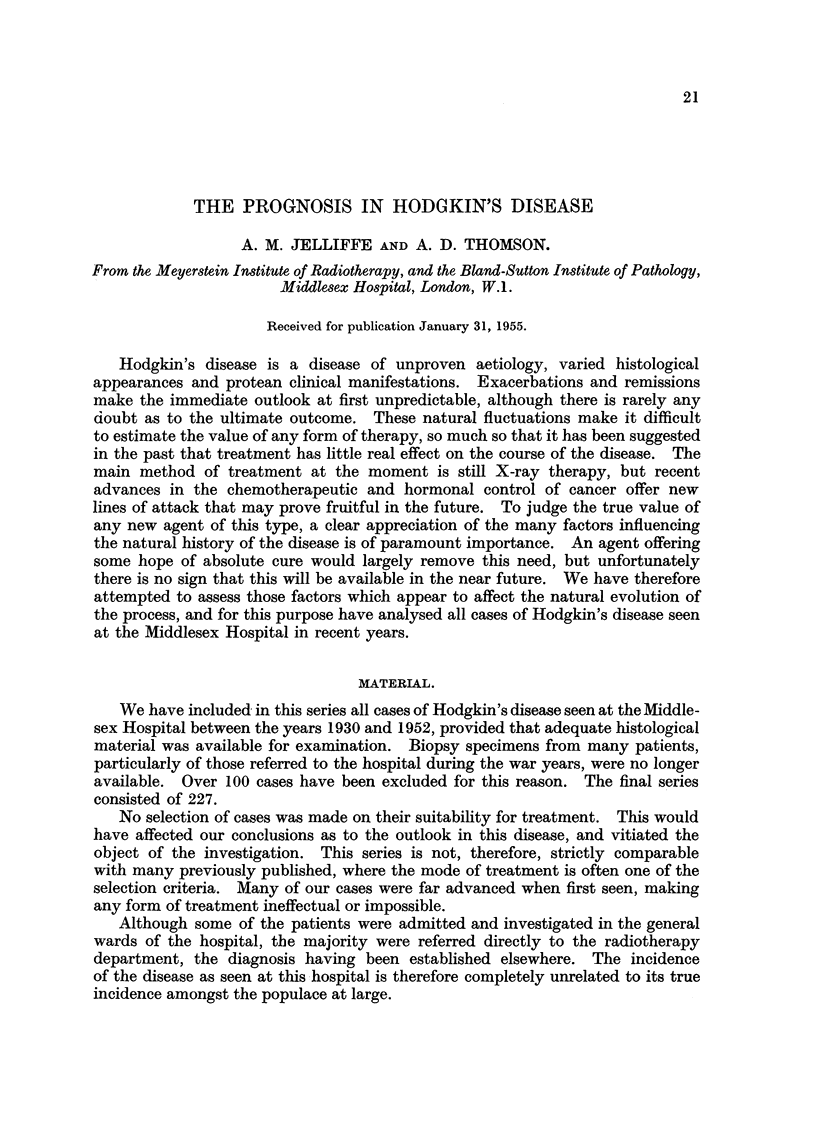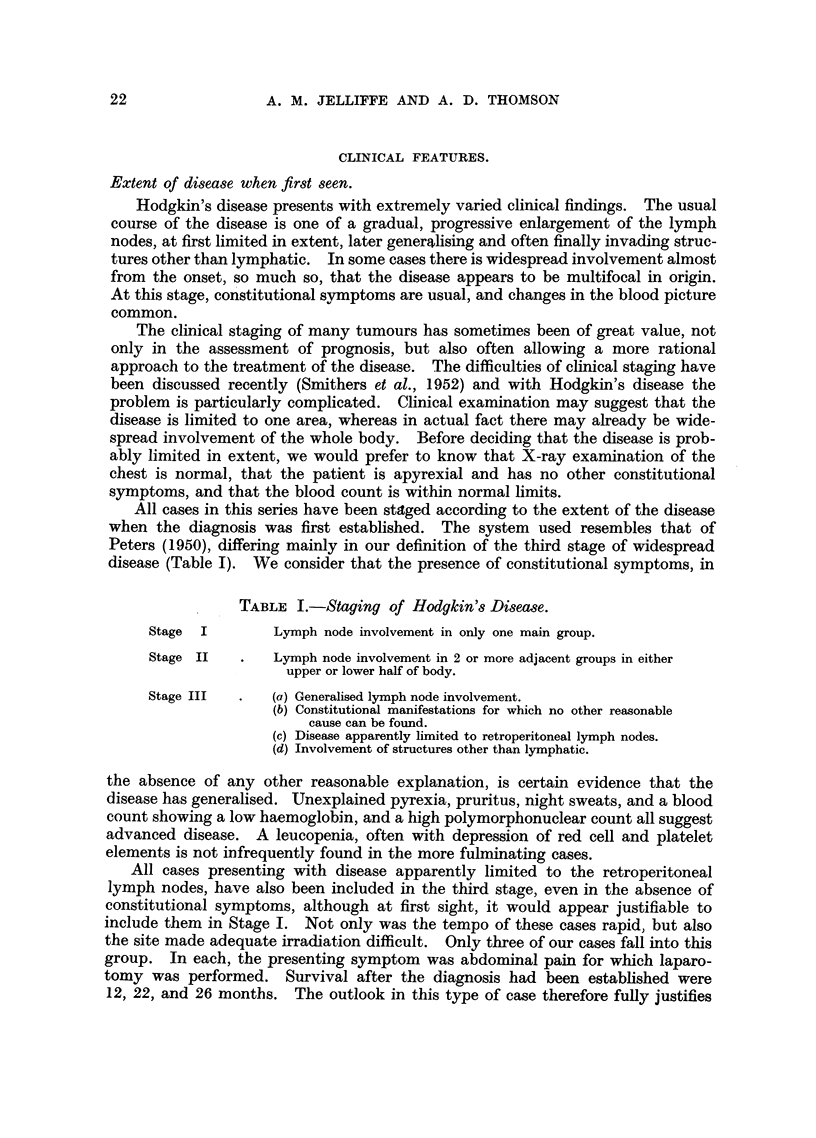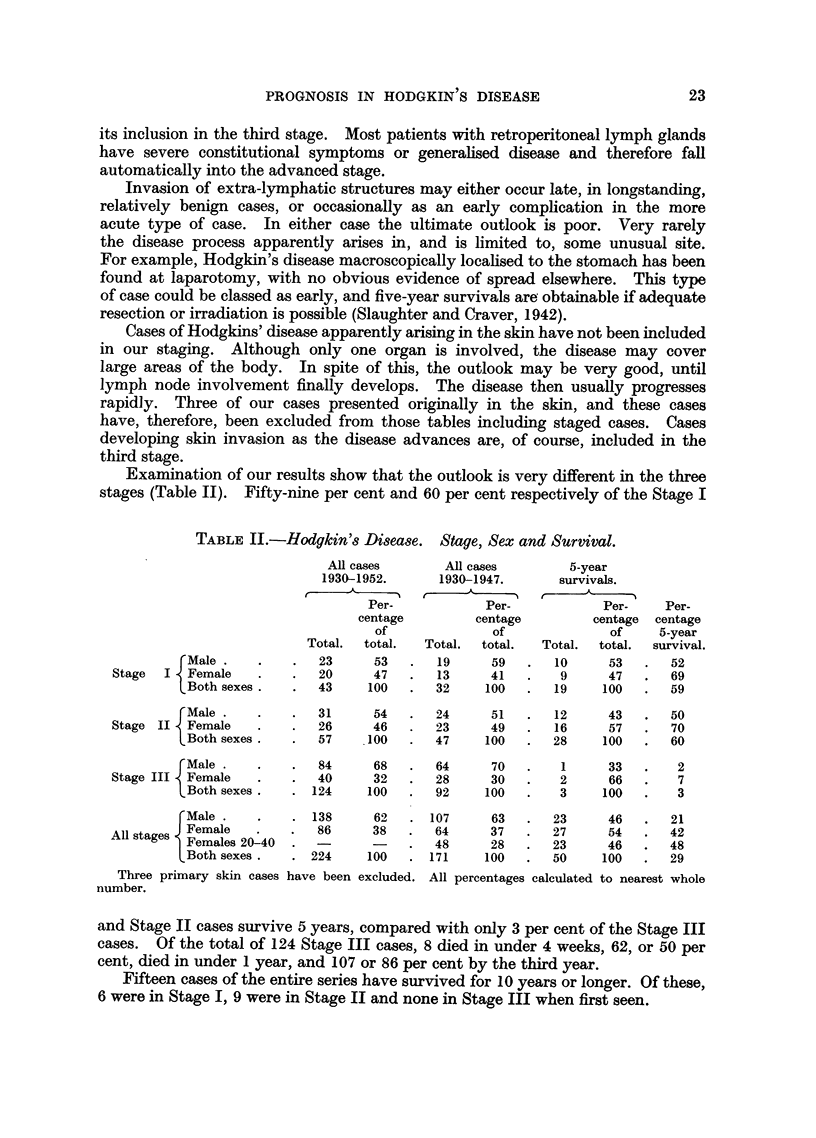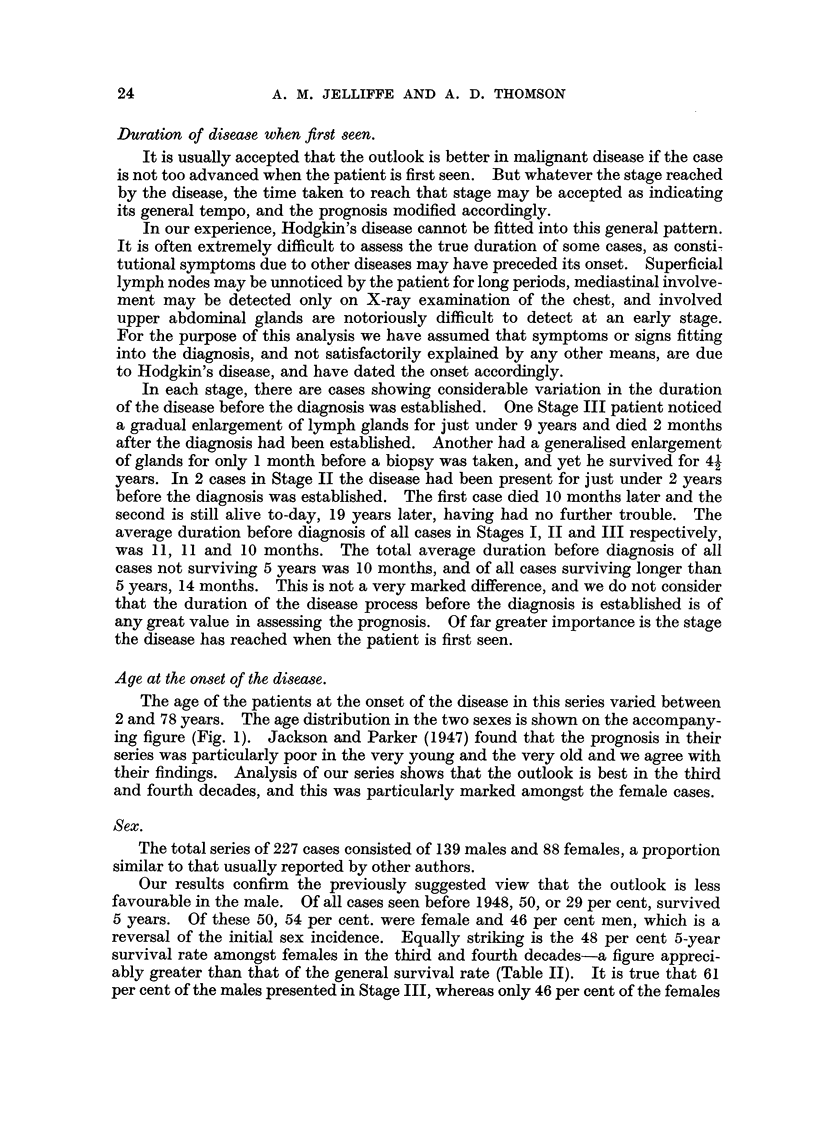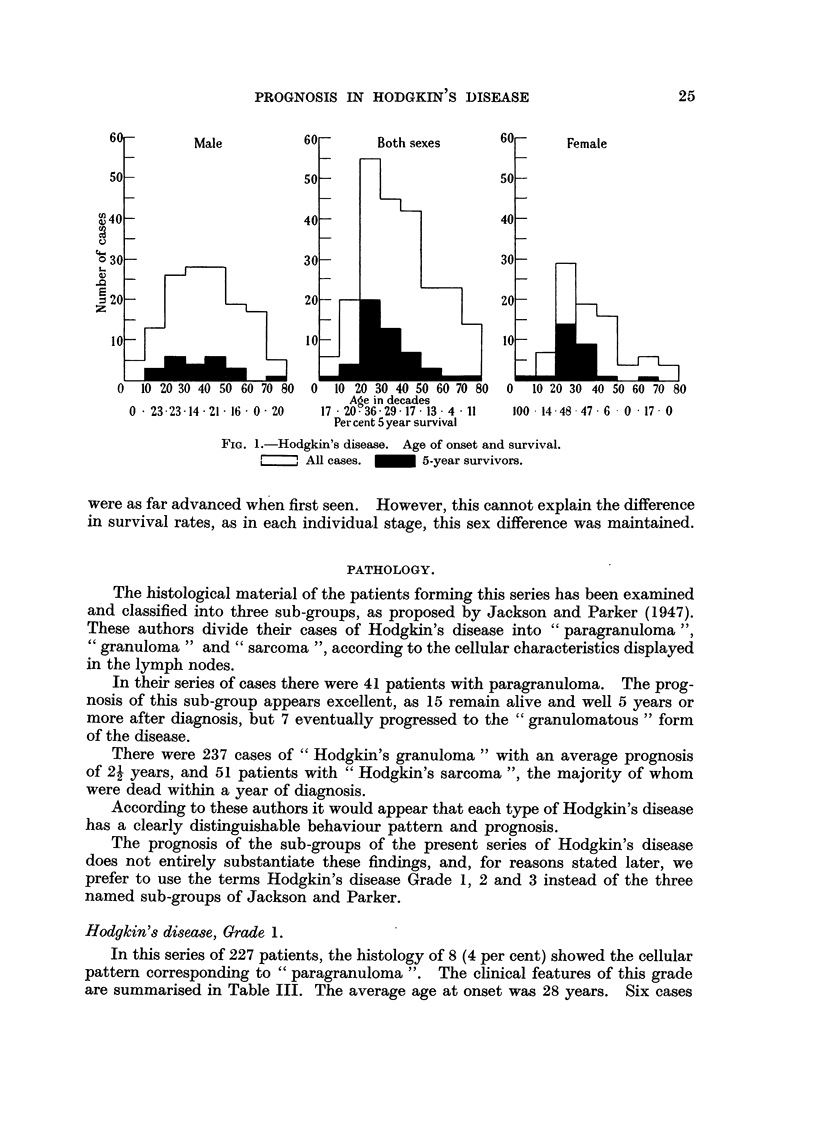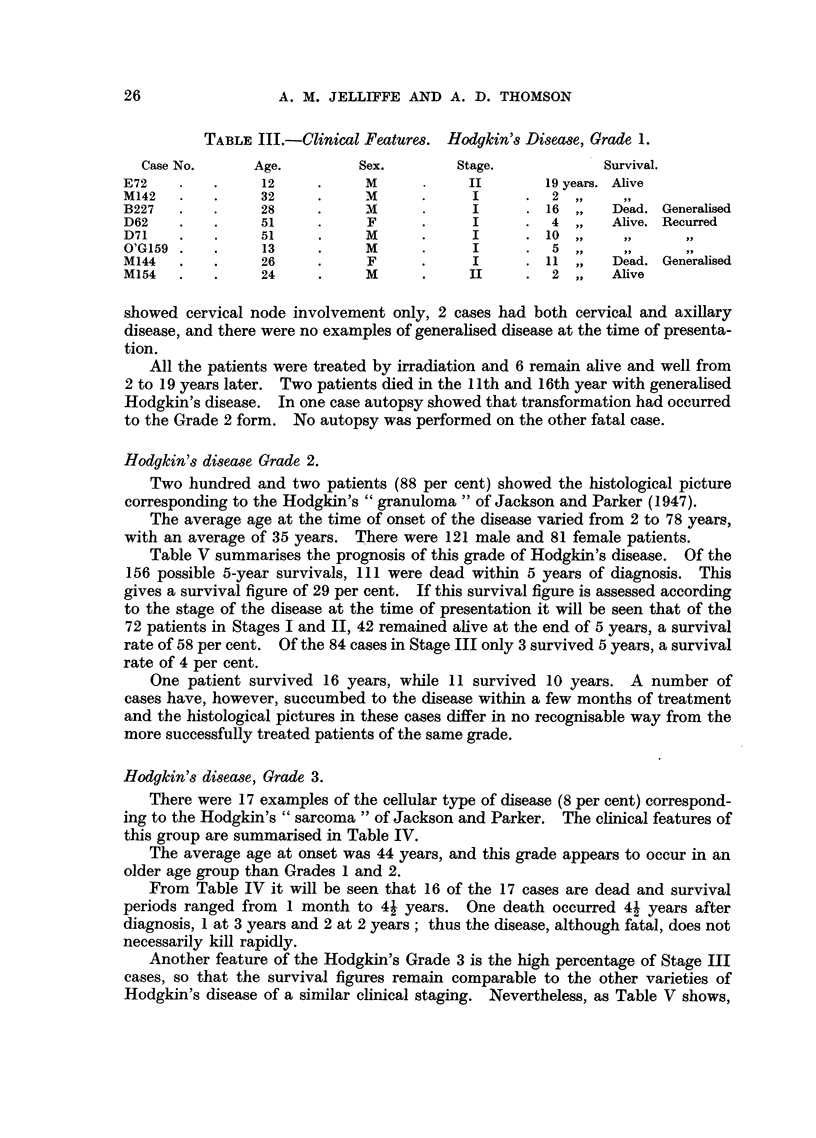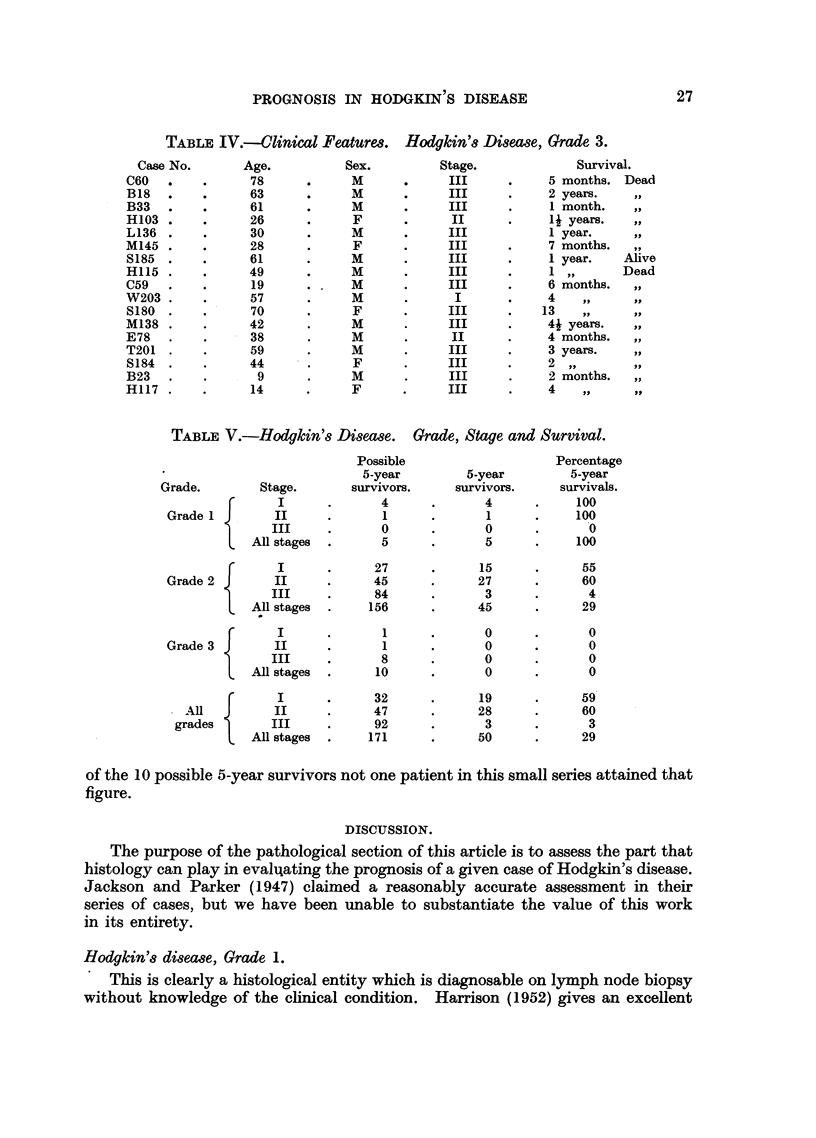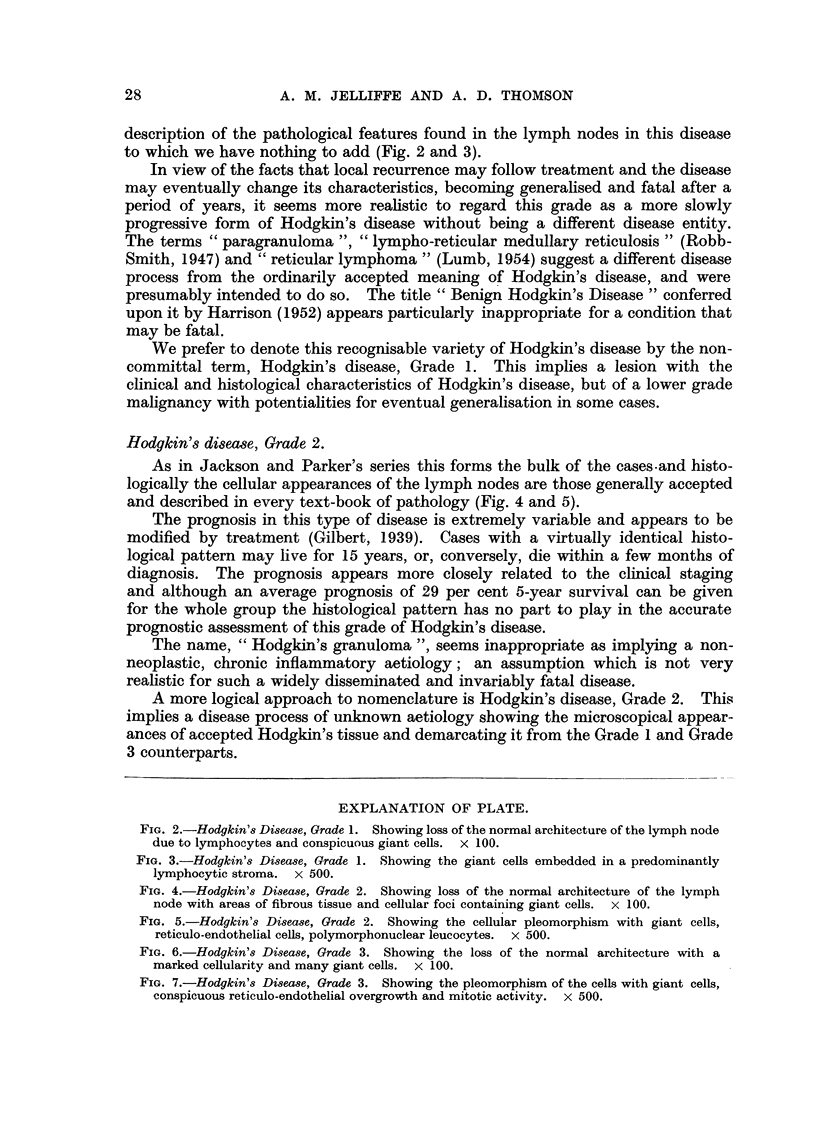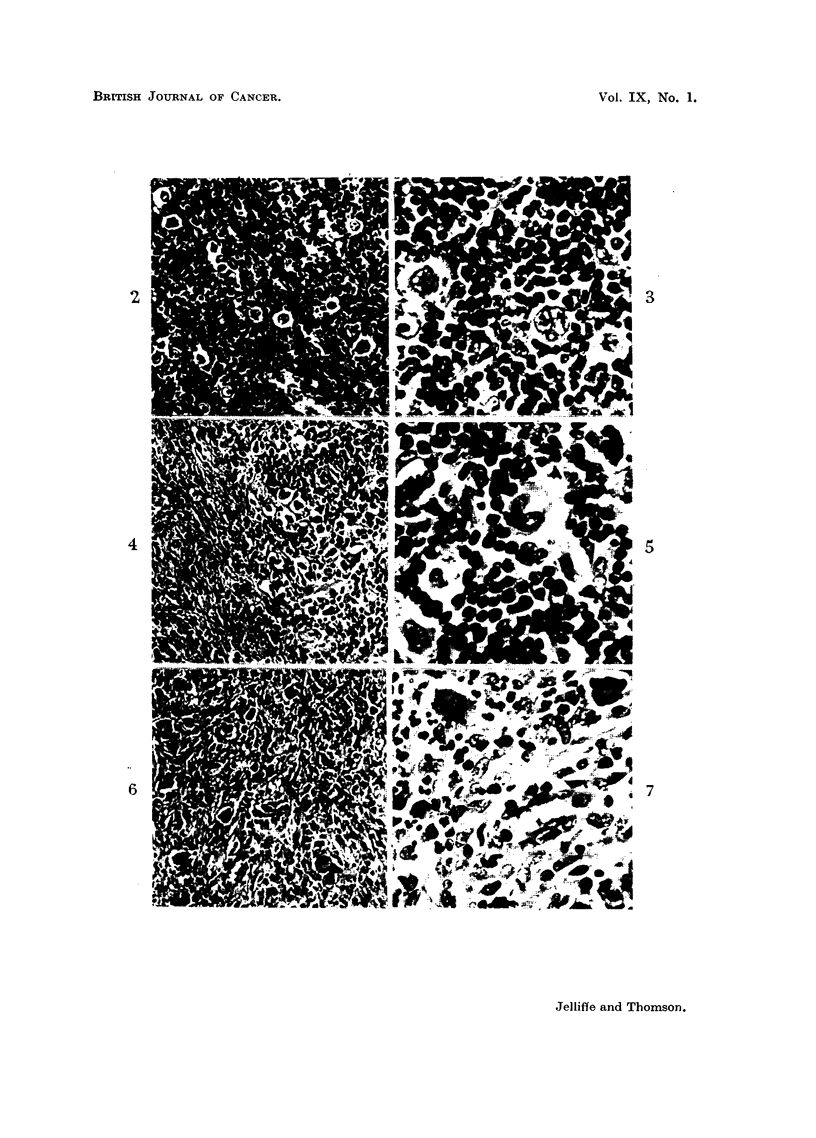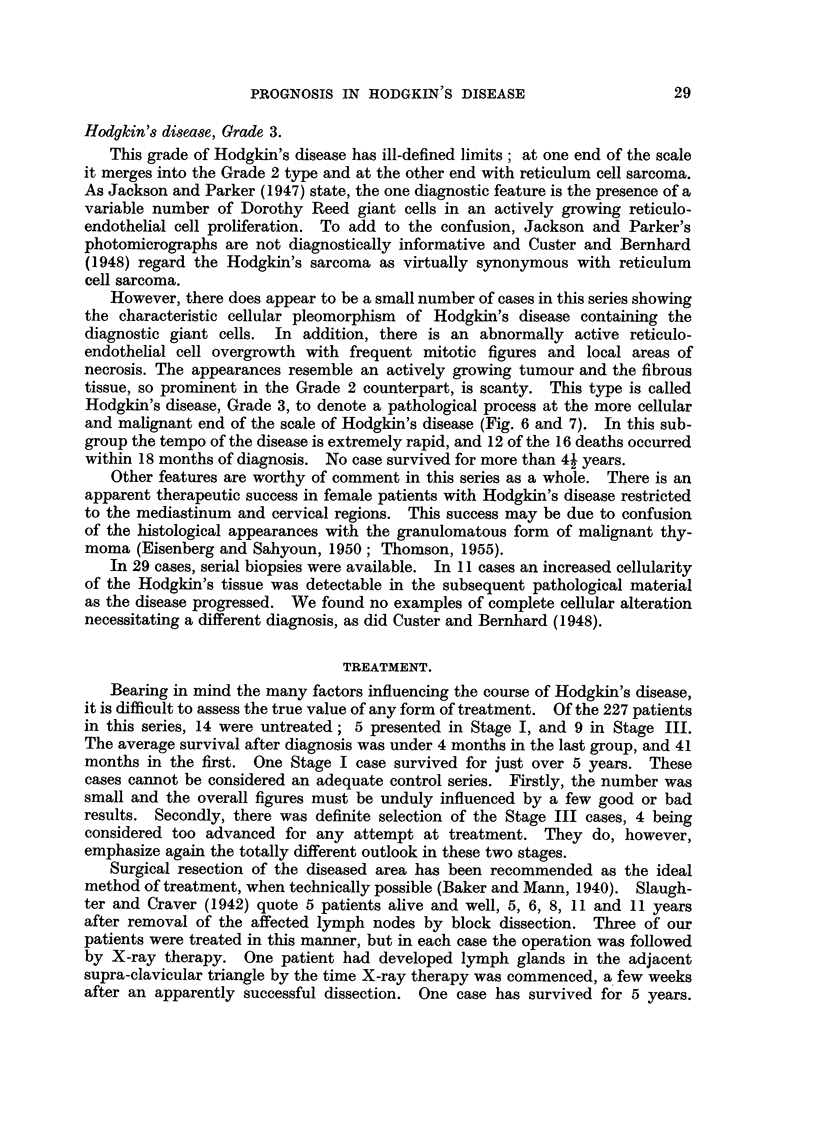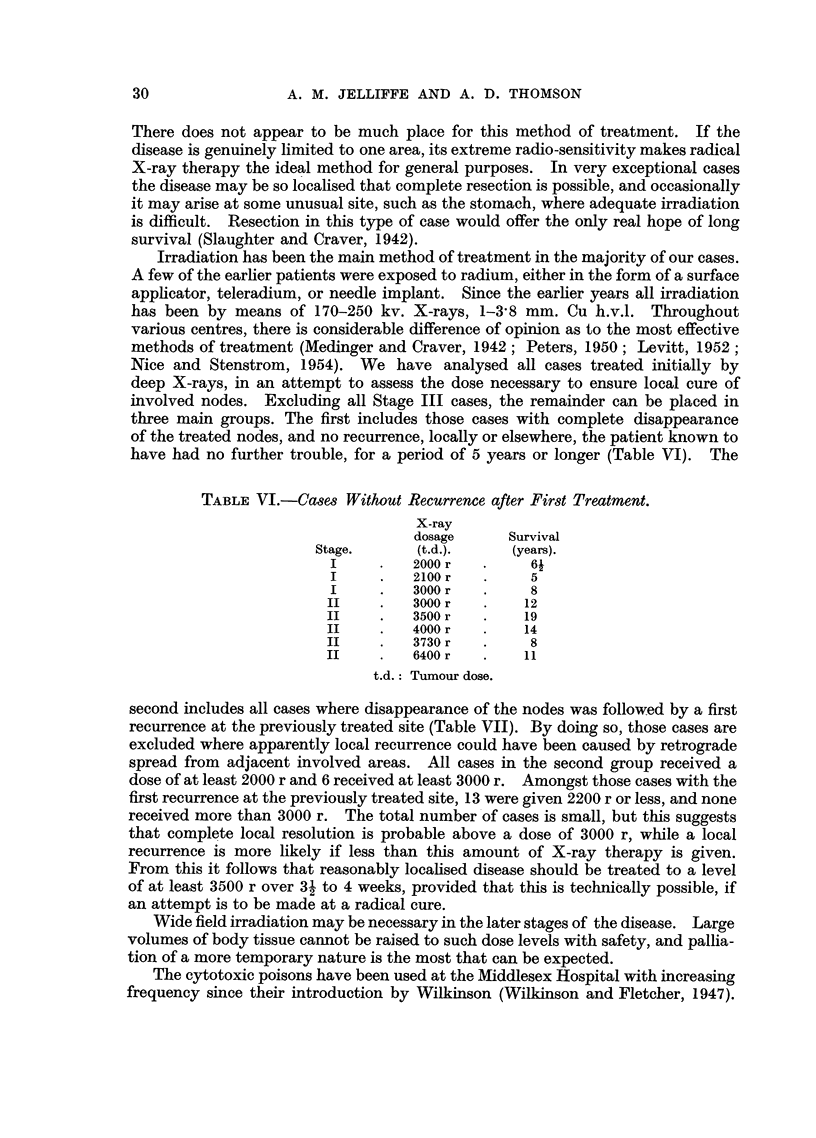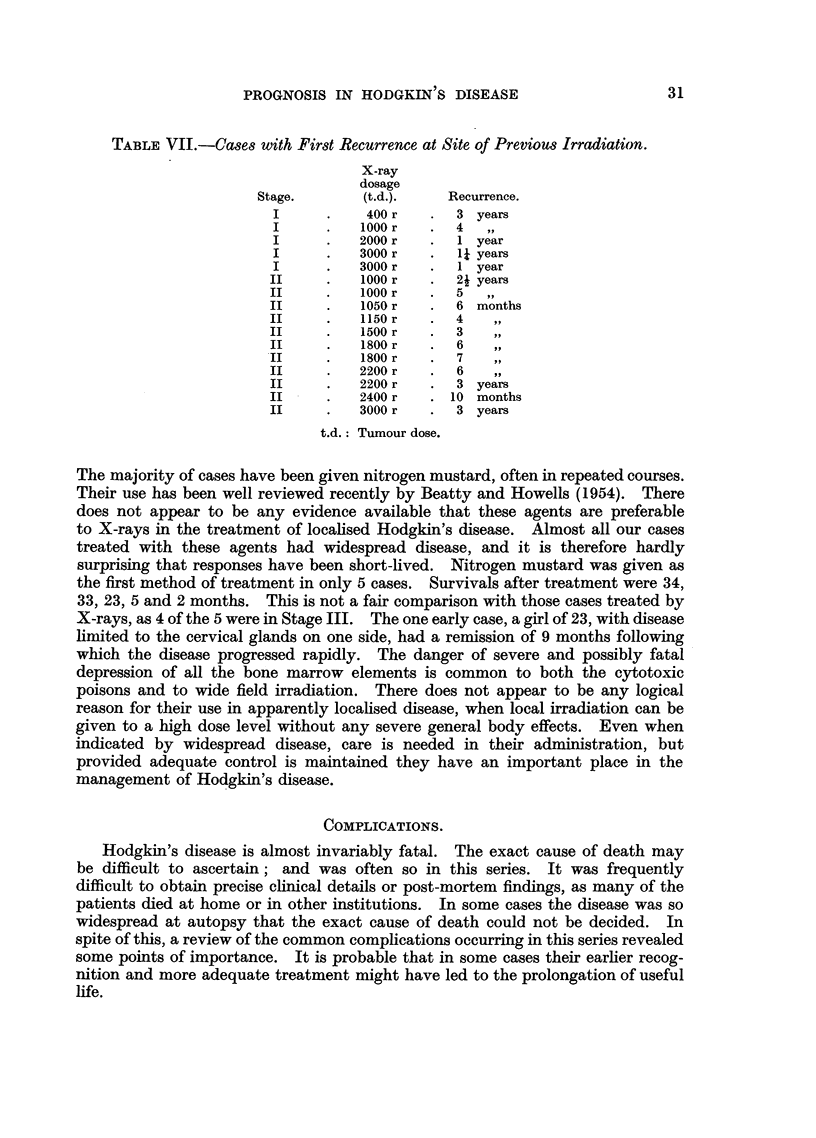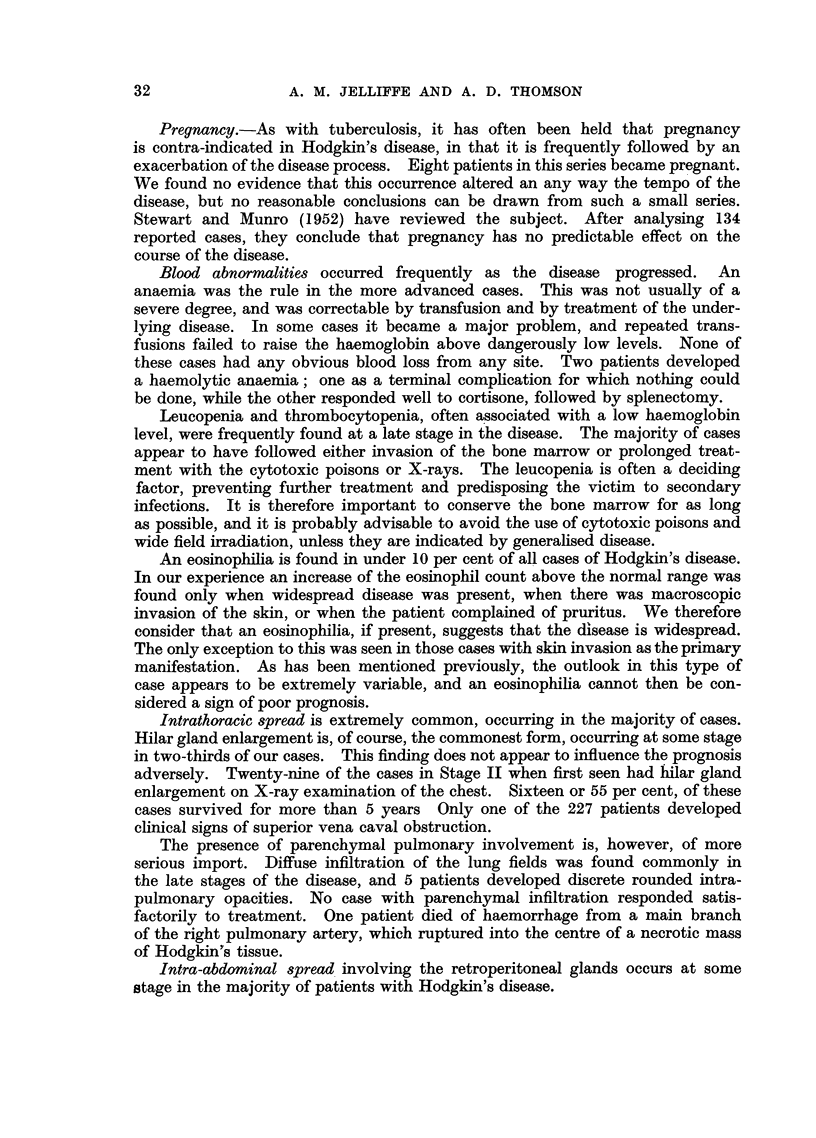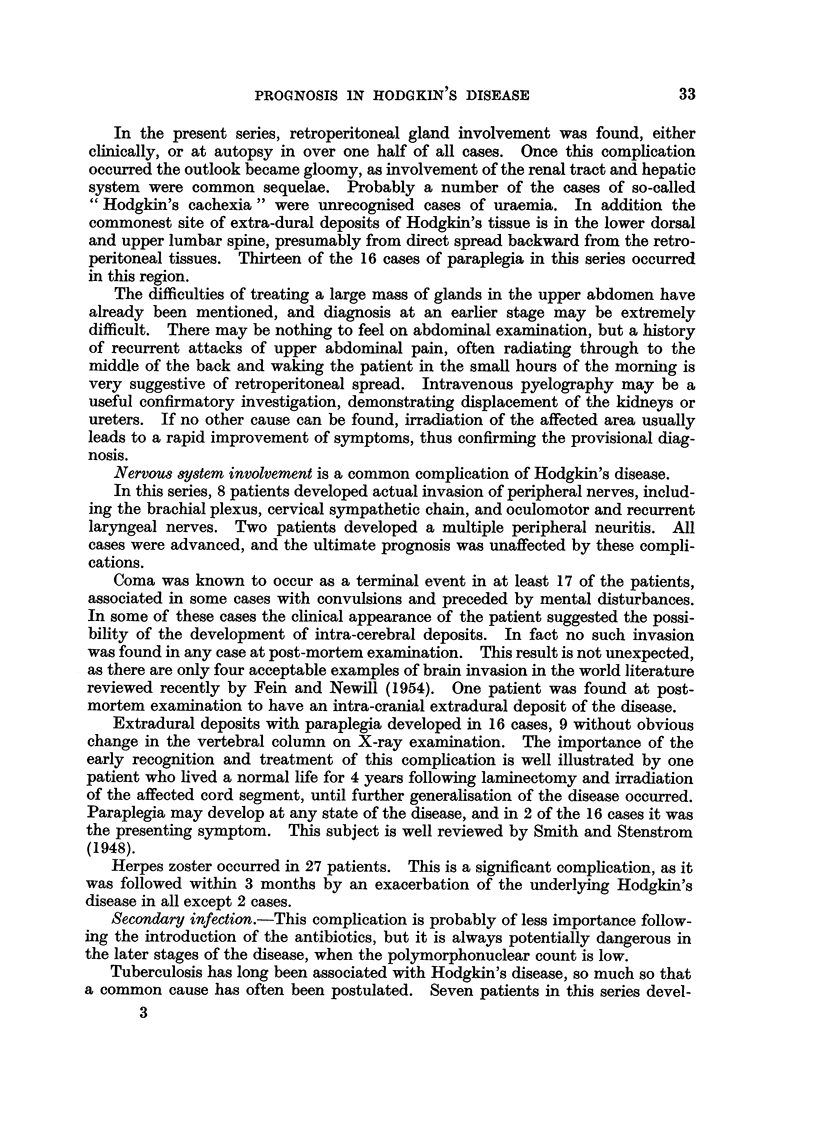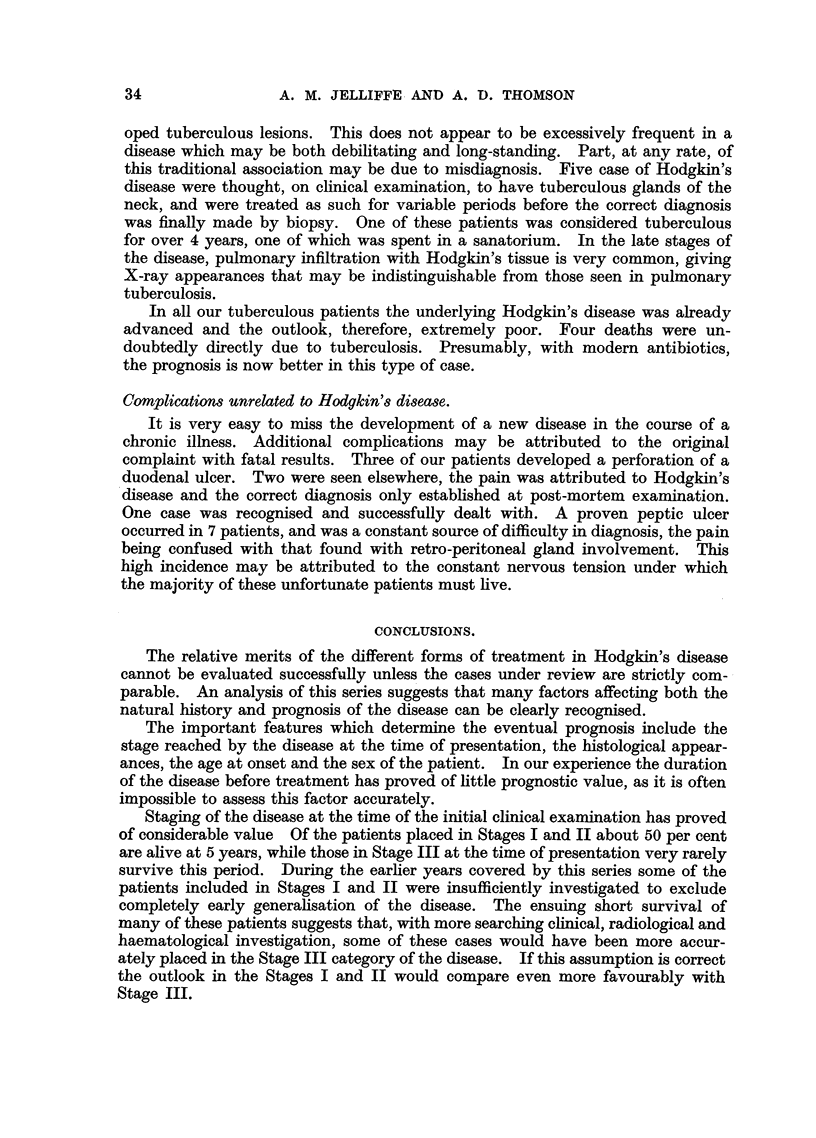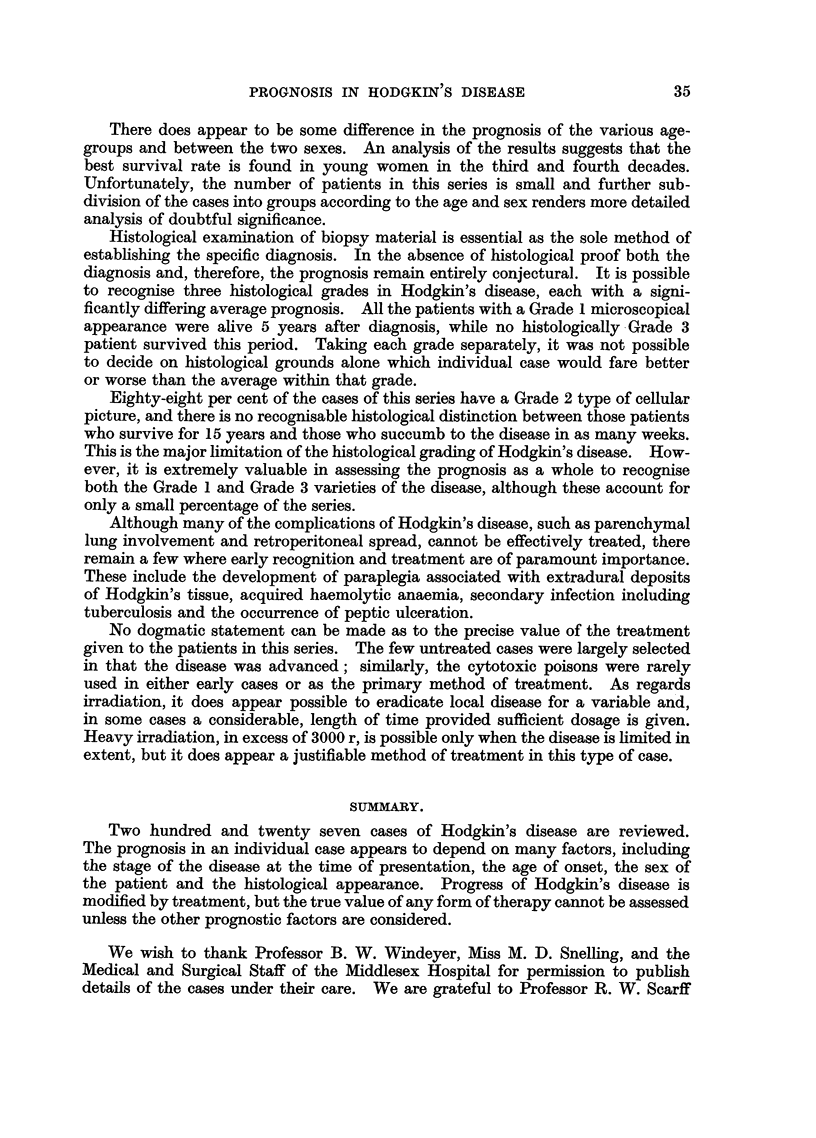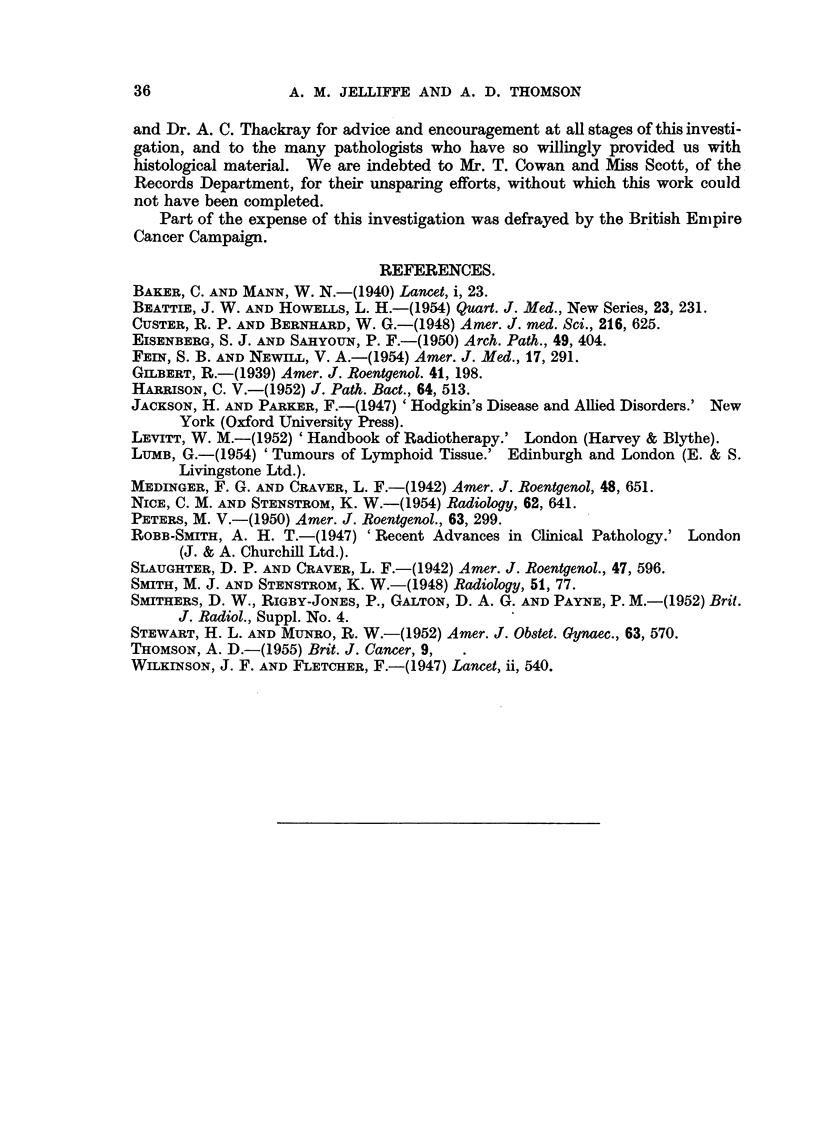# The Prognosis in Hodgkin's Disease

**DOI:** 10.1038/bjc.1955.3

**Published:** 1955-03

**Authors:** A. M. Jelliffe, A. D. Thomson

## Abstract

**Images:**


### THE PROGNOSIS IN HODGKIN'S DISEASE

A. M. JELLIFFE AND A. D. THOMSON.

*From the Meyerstein Institute of Radiotherapy, and the Bland-Sutton Institute of Pathology, Middlesex Hospital, London, W.1.*

Received for publication January 31, 1955.

Hodgkin's disease is a disease of unproven aetiology, varied histological appearances and protean clinical manifestations. Exacerbations and remissions make the immediate outlook at first unpredictable, although there is rarely any doubt as to the ultimate outcome. These natural fluctuations make it difficult to estimate the value of any form of therapy, so much so that it has been suggested in the past that treatment has little real effect on the course of the disease. The main method of treatment at the moment is still X-ray therapy, but recent advances in the chemotherapeutic and hormonal control of cancer offer new lines of attack that may prove fruitful in the future. To judge the true value of any new agent of this type, a clear appreciation of the many factors influencing the natural history of the disease is of paramount importance. An agent offering some hope of absolute cure would largely remove this need, but unfortunately there is no sign that this will be available in the near future. We have therefore attempted to assess those factors which appear to affect the natural evolution of the process, and for this purpose have analysed all cases of Hodgkin's disease seen at the Middlesex Hospital in recent years.

### MATERIAL.

We have included in this series all cases of Hodgkin's disease seen at the Middlesex Hospital between the years 1930 and 1952, provided that adequate histological material was available for examination. Biopsy specimens from many patients, particularly of those referred to the hospital during the war years, were no longer available. Over 100 cases have been excluded for this reason. The final series consisted of 227.

No selection of cases was made on their suitability for treatment. This would have affected our conclusions as to the outlook in this disease, and vitiated the object of the investigation. This series is not, therefore, strictly comparable with many previously published, where the mode of treatment is often one of the selection criteria. Many of our cases were far advanced when first seen, making any form of treatment ineffectual or impossible.

Although some of the patients were admitted and investigated in the general wards of the hospital, the majority were referred directly to the radiotherapy department, the diagnosis having been established elsewhere. The incidence of the disease as seen at this hospital is therefore completely unrelated to its true incidence amongst the populace at large.

### CLINICAL FEATURES.

*Extent of disease when first seen.*

Hodgkin's disease presents with extremely varied clinical findings. The usual course of the disease is one of a gradual, progressive enlargement of the lymph nodes, at first limited in extent, later generalising and often finally invading structures other than lymphatic. In some cases there is widespread involvement almost from the onset, so much so, that the disease appears to be multifocal in origin. At this stage, constitutional symptoms are usual, and changes in the blood picture common.

The clinical staging of many tumours has sometimes been of great value, not only in the assessment of prognosis, but also often allowing a more rational approach to the treatment of the disease. The difficulties of clinical staging have been discussed recently (Smithers *et al.*, 1952) and with Hodgkin's disease the problem is particularly complicated. Clinical examination may suggest that the disease is limited to one area, whereas in actual fact there may already be widespread involvement of the whole body. Before deciding that the disease is probably limited in extent, we would prefer to know that X-ray examination of the chest is normal, that the patient is apyrexial and has no other constitutional symptoms, and that the blood count is within normal limits.

All cases in this series have been staged according to the extent of the disease when the diagnosis was first established. The system used resembles that of Peters (1950), differing mainly in our definition of the third stage of widespread disease (Table I). We consider that the presence of constitutional symptoms, in

TABLE I.—*Staging of Hodgkin's Disease.*

| | |
|---|---|
| Stage I | Lymph node involvement in only one main group. |
| Stage II . | Lymph node involvement in 2 or more adjacent groups in either upper or lower half of body. |
| Stage III . | (*a*) Generalised lymph node involvement.<br>(*b*) Constitutional manifestations for which no other reasonable cause can be found.<br>(*c*) Disease apparently limited to retroperitoneal lymph nodes.<br>(*d*) Involvement of structures other than lymphatic. |

the absence of any other reasonable explanation, is certain evidence that the disease has generalised. Unexplained pyrexia, pruritus, night sweats, and a blood count showing a low haemoglobin, and a high polymorphonuclear count all suggest advanced disease. A leucopenia, often with depression of red cell and platelet elements is not infrequently found in the more fulminating cases.

All cases presenting with disease apparently limited to the retroperitoneal lymph nodes, have also been included in the third stage, even in the absence of constitutional symptoms, although at first sight, it would appear justifiable to include them in Stage I. Not only was the tempo of these cases rapid, but also the site made adequate irradiation difficult. Only three of our cases fall into this group. In each, the presenting symptom was abdominal pain for which laparotomy was performed. Survival after the diagnosis had been established were 12, 22, and 26 months. The outlook in this type of case therefore fully justifies its inclusion in the third stage. Most patients with retroperitoneal lymph glands have severe constitutional symptoms or generalised disease and therefore fall automatically into the advanced stage.

Invasion of extra-lymphatic structures may either occur late, in longstanding, relatively benign cases, or occasionally as an early complication in the more acute type of case. In either case the ultimate outlook is poor. Very rarely the disease process apparently arises in, and is limited to, some unusual site. For example, Hodgkin's disease macroscopically localised to the stomach has been found at laparotomy, with no obvious evidence of spread elsewhere. This type of case could be classed as early, and five-year survivals are obtainable if adequate resection or irradiation is possible (Slaughter and Craver, 1942).

Cases of Hodgkins' disease apparently arising in the skin have not been included in our staging. Although only one organ is involved, the disease may cover large areas of the body. In spite of this, the outlook may be very good, until lymph node involvement finally develops. The disease then usually progresses rapidly. Three of our cases presented originally in the skin, and these cases have, therefore, been excluded from those tables including staged cases. Cases developing skin invasion as the disease advances are, of course, included in the third stage.

Examination of our results show that the outlook is very different in the three stages (Table II). Fifty-nine per cent and 60 per cent respectively of the Stage I

TABLE II.—*Hodgkin's Disease. Stage, Sex and Survival.*

| | | All cases 1930–1952. | | All cases 1930–1947. | | 5-year survivals. | | |
|---|---|---|---|---|---|---|---|---|
| | | Total. | Percentage of total. | Total. | Percentage of total. | Total. | Percentage of total. | Percentage 5-year survival. |
| Stage I | Male | 23 | 53 | 19 | 59 | 10 | 53 | 52 |
| | Female | 20 | 47 | 13 | 41 | 9 | 47 | 69 |
| | Both sexes | 43 | 100 | 32 | 100 | 19 | 100 | 59 |
| Stage II | Male | 31 | 54 | 24 | 51 | 12 | 43 | 50 |
| | Female | 26 | 46 | 23 | 49 | 16 | 57 | 70 |
| | Both sexes | 57 | 100 | 47 | 100 | 28 | 100 | 60 |
| Stage III | Male | 84 | 68 | 64 | 70 | 1 | 33 | 2 |
| | Female | 40 | 32 | 28 | 30 | 2 | 66 | 7 |
| | Both sexes | 124 | 100 | 92 | 100 | 3 | 100 | 3 |
| All stages | Male | 138 | 62 | 107 | 63 | 23 | 46 | 21 |
| | Female | 86 | 38 | 64 | 37 | 27 | 54 | 42 |
| | Females 20–40 | — | — | 48 | 28 | 23 | 46 | 48 |
| | Both sexes | 224 | 100 | 171 | 100 | 50 | 100 | 29 |

Three primary skin cases have been excluded. All percentages calculated to nearest whole number.

and Stage II cases survive 5 years, compared with only 3 per cent of the Stage III cases. Of the total of 124 Stage III cases, 8 died in under 4 weeks, 62, or 50 per cent, died in under 1 year, and 107 or 86 per cent by the third year.

Fifteen cases of the entire series have survived for 10 years or longer. Of these, 6 were in Stage I, 9 were in Stage II and none in Stage III when first seen.

*Duration of disease when first seen.*

It is usually accepted that the outlook is better in malignant disease if the case is not too advanced when the patient is first seen. But whatever the stage reached by the disease, the time taken to reach that stage may be accepted as indicating its general tempo, and the prognosis modified accordingly.

In our experience, Hodgkin's disease cannot be fitted into this general pattern. It is often extremely difficult to assess the true duration of some cases, as constitutional symptoms due to other diseases may have preceded its onset. Superficial lymph nodes may be unnoticed by the patient for long periods, mediastinal involvement may be detected only on X-ray examination of the chest, and involved upper abdominal glands are notoriously difficult to detect at an early stage. For the purpose of this analysis we have assumed that symptoms or signs fitting into the diagnosis, and not satisfactorily explained by any other means, are due to Hodgkin's disease, and have dated the onset accordingly.

In each stage, there are cases showing considerable variation in the duration of the disease before the diagnosis was established. One Stage III patient noticed a gradual enlargement of lymph glands for just under 9 years and died 2 months after the diagnosis had been established. Another had a generalised enlargement of glands for only 1 month before a biopsy was taken, and yet he survived for 4½ years. In 2 cases in Stage II the disease had been present for just under 2 years before the diagnosis was established. The first case died 10 months later and the second is still alive to-day, 19 years later, having had no further trouble. The average duration before diagnosis of all cases in Stages I, II and III respectively, was 11, 11 and 10 months. The total average duration before diagnosis of all cases not surviving 5 years was 10 months, and of all cases surviving longer than 5 years, 14 months. This is not a very marked difference, and we do not consider that the duration of the disease process before the diagnosis is established is of any great value in assessing the prognosis. Of far greater importance is the stage the disease has reached when the patient is first seen.

*Age at the onset of the disease.*

The age of the patients at the onset of the disease in this series varied between 2 and 78 years. The age distribution in the two sexes is shown on the accompanying figure (Fig. 1). Jackson and Parker (1947) found that the prognosis in their series was particularly poor in the very young and the very old and we agree with their findings. Analysis of our series shows that the outlook is best in the third and fourth decades, and this was particularly marked amongst the female cases.

*Sex.*

The total series of 227 cases consisted of 139 males and 88 females, a proportion similar to that usually reported by other authors.

Our results confirm the previously suggested view that the outlook is less favourable in the male. Of all cases seen before 1948, 50, or 29 per cent, survived 5 years. Of these 50, 54 per cent. were female and 46 per cent men, which is a reversal of the initial sex incidence. Equally striking is the 48 per cent 5-year survival rate amongst females in the third and fourth decades—a figure appreciably greater than that of the general survival rate (Table II). It is true that 61 per cent of the males presented in Stage III, whereas only 46 per cent of the females

FIG. 1.—Hodgkin's disease. Age of onset and survival.
All cases. 5-year survivors.

were as far advanced when first seen. However, this cannot explain the difference in survival rates, as in each individual stage, this sex difference was maintained.

### PATHOLOGY.

The histological material of the patients forming this series has been examined and classified into three sub-groups, as proposed by Jackson and Parker (1947). These authors divide their cases of Hodgkin's disease into " paragranuloma ", " granuloma " and " sarcoma ", according to the cellular characteristics displayed in the lymph nodes.

In their series of cases there were 41 patients with paragranuloma. The prognosis of this sub-group appears excellent, as 15 remain alive and well 5 years or more after diagnosis, but 7 eventually progressed to the " granulomatous " form of the disease.

There were 237 cases of " Hodgkin's granuloma " with an average prognosis of 2½ years, and 51 patients with " Hodgkin's sarcoma ", the majority of whom were dead within a year of diagnosis.

According to these authors it would appear that each type of Hodgkin's disease has a clearly distinguishable behaviour pattern and prognosis.

The prognosis of the sub-groups of the present series of Hodgkin's disease does not entirely substantiate these findings, and, for reasons stated later, we prefer to use the terms Hodgkin's disease Grade 1, 2 and 3 instead of the three named sub-groups of Jackson and Parker.

*Hodgkin's disease, Grade* 1.

In this series of 227 patients, the histology of 8 (4 per cent) showed the cellular pattern corresponding to " paragranuloma ". The clinical features of this grade are summarised in Table III. The average age at onset was 28 years. Six cases

TABLE III.—*Clinical Features. Hodgkin's Disease, Grade* 1.

| Case No. | Age. | Sex. | Stage. | Survival. | | |
|---|---|---|---|---|---|---|
| E72 | 12 | M | II | 19 years. | Alive | |
| M142 | 32 | M | I | 2 ,, | ,, | |
| B227 | 28 | M | I | 16 ,, | Dead. | Generalised |
| D62 | 51 | F | I | 4 ,, | Alive. | Recurred |
| D71 | 51 | M | I | 10 ,, | ,, | ,, |
| O'G159 | 13 | M | I | 5 ,, | ,, | ,, |
| M144 | 26 | F | I | 11 ,, | Dead. | Generalised |
| M154 | 24 | M | II | 2 ,, | Alive | |

showed cervical node involvement only, 2 cases had both cervical and axillary disease, and there were no examples of generalised disease at the time of presentation.

All the patients were treated by irradiation and 6 remain alive and well from 2 to 19 years later. Two patients died in the 11th and 16th year with generalised Hodgkin's disease. In one case autopsy showed that transformation had occurred to the Grade 2 form. No autopsy was performed on the other fatal case.

*Hodgkin's disease Grade* 2.

Two hundred and two patients (88 per cent) showed the histological picture corresponding to the Hodgkin's " granuloma " of Jackson and Parker (1947).

The average age at the time of onset of the disease varied from 2 to 78 years, with an average of 35 years. There were 121 male and 81 female patients.

Table V summarises the prognosis of this grade of Hodgkin's disease. Of the 156 possible 5-year survivals, 111 were dead within 5 years of diagnosis. This gives a survival figure of 29 per cent. If this survival figure is assessed according to the stage of the disease at the time of presentation it will be seen that of the 72 patients in Stages I and II, 42 remained alive at the end of 5 years, a survival rate of 58 per cent. Of the 84 cases in Stage III only 3 survived 5 years, a survival rate of 4 per cent.

One patient survived 16 years, while 11 survived 10 years. A number of cases have, however, succumbed to the disease within a few months of treatment and the histological pictures in these cases differ in no recognisable way from the more successfully treated patients of the same grade.

*Hodgkin's disease, Grade* 3.

There were 17 examples of the cellular type of disease (8 per cent) corresponding to the Hodgkin's " sarcoma " of Jackson and Parker. The clinical features of this group are summarised in Table IV.

The average age at onset was 44 years, and this grade appears to occur in an older age group than Grades 1 and 2.

From Table IV it will be seen that 16 of the 17 cases are dead and survival periods ranged from 1 month to $4\frac{1}{2}$ years. One death occurred $4\frac{1}{2}$ years after diagnosis, 1 at 3 years and 2 at 2 years ; thus the disease, although fatal, does not necessarily kill rapidly.

Another feature of the Hodgkin's Grade 3 is the high percentage of Stage III cases, so that the survival figures remain comparable to the other varieties of Hodgkin's disease of a similar clinical staging. Nevertheless, as Table V shows,

TABLE IV.—*Clinical Features. Hodgkin's Disease, Grade* 3.

| Case No. | Age. | Sex. | Stage. | Survival. | |
|---|---|---|---|---|---|
| C60 | 78 | M | III | 5 months. | Dead |
| B18 | 63 | M | III | 2 years. | ,, |
| B33 | 61 | M | III | 1 month. | ,, |
| H103 | 26 | F | II | 1½ years. | ,, |
| L136 | 30 | M | III | 1 year. | ,, |
| M145 | 28 | F | III | 7 months. | ,, |
| S185 | 61 | M | III | 1 year. | Alive |
| H115 | 49 | M | III | 1 ,, | Dead |
| C59 | 19 | M | III | 6 months. | ,, |
| W203 | 57 | M | I | 4 ,, | ,, |
| S180 | 70 | F | III | 13 ,, | ,, |
| M138 | 42 | M | III | 4½ years. | ,, |
| E78 | 38 | M | II | 4 months. | ,, |
| T201 | 59 | M | III | 3 years. | ,, |
| S184 | 44 | F | III | 2 ,, | ,, |
| B23 | 9 | M | III | 2 months. | ,, |
| H117 | 14 | F | III | 4 ,, | ,, |

TABLE V.—*Hodgkin's Disease. Grade, Stage and Survival.*

| Grade. | Stage. | Possible 5-year survivors. | 5-year survivors. | Percentage 5-year survivals. |
|---|---|---|---|---|
| Grade 1 | I | 4 | 4 | 100 |
| | II | 1 | 1 | 100 |
| | III | 0 | 0 | 0 |
| | All stages | 5 | 5 | 100 |
| Grade 2 | I | 27 | 15 | 55 |
| | II | 45 | 27 | 60 |
| | III | 84 | 3 | 4 |
| | All stages | 156 | 45 | 29 |
| Grade 3 | I | 1 | 0 | 0 |
| | II | 1 | 0 | 0 |
| | III | 8 | 0 | 0 |
| | All stages | 10 | 0 | 0 |
| All grades | I | 32 | 19 | 59 |
| | II | 47 | 28 | 60 |
| | III | 92 | 3 | 3 |
| | All stages | 171 | 50 | 29 |

of the 10 possible 5-year survivors not one patient in this small series attained that figure.

DISCUSSION.

The purpose of the pathological section of this article is to assess the part that histology can play in evaluating the prognosis of a given case of Hodgkin's disease. Jackson and Parker (1947) claimed a reasonably accurate assessment in their series of cases, but we have been unable to substantiate the value of this work in its entirety.

*Hodgkin's disease, Grade* 1.

This is clearly a histological entity which is diagnosable on lymph node biopsy without knowledge of the clinical condition. Harrison (1952) gives an excellent description of the pathological features found in the lymph nodes in this disease to which we have nothing to add (Fig. 2 and 3).

In view of the facts that local recurrence may follow treatment and the disease may eventually change its characteristics, becoming generalised and fatal after a period of years, it seems more realistic to regard this grade as a more slowly progressive form of Hodgkin's disease without being a different disease entity. The terms " paragranuloma ", " lympho-reticular medullary reticulosis " (Robb-Smith, 1947) and " reticular lymphoma " (Lumb, 1954) suggest a different disease process from the ordinarily accepted meaning of Hodgkin's disease, and were presumably intended to do so. The title " Benign Hodgkin's Disease " conferred upon it by Harrison (1952) appears particularly inappropriate for a condition that may be fatal.

We prefer to denote this recognisable variety of Hodgkin's disease by the non-committal term, Hodgkin's disease, Grade 1. This implies a lesion with the clinical and histological characteristics of Hodgkin's disease, but of a lower grade malignancy with potentialities for eventual generalisation in some cases.

*Hodgkin's disease, Grade 2.*

As in Jackson and Parker's series this forms the bulk of the cases and histologically the cellular appearances of the lymph nodes are those generally accepted and described in every text-book of pathology (Fig. 4 and 5).

The prognosis in this type of disease is extremely variable and appears to be modified by treatment (Gilbert, 1939). Cases with a virtually identical histological pattern may live for 15 years, or, conversely, die within a few months of diagnosis. The prognosis appears more closely related to the clinical staging and although an average prognosis of 29 per cent 5-year survival can be given for the whole group the histological pattern has no part to play in the accurate prognostic assessment of this grade of Hodgkin's disease.

The name, " Hodgkin's granuloma ", seems inappropriate as implying a non-neoplastic, chronic inflammatory aetiology; an assumption which is not very realistic for such a widely disseminated and invariably fatal disease.

A more logical approach to nomenclature is Hodgkin's disease, Grade 2. This implies a disease process of unknown aetiology showing the microscopical appearances of accepted Hodgkin's tissue and demarcating it from the Grade 1 and Grade 3 counterparts.

---

EXPLANATION OF PLATE.

FIG. 2.—*Hodgkin's Disease, Grade* 1. Showing loss of the normal architecture of the lymph node due to lymphocytes and conspicuous giant cells. × 100.

FIG. 3.—*Hodgkin's Disease, Grade* 1. Showing the giant cells embedded in a predominantly lymphocytic stroma. × 500.

FIG. 4.—*Hodgkin's Disease, Grade* 2. Showing loss of the normal architecture of the lymph node with areas of fibrous tissue and cellular foci containing giant cells. × 100.

FIG. 5.—*Hodgkin's Disease, Grade* 2. Showing the cellular pleomorphism with giant cells, reticulo-endothelial cells, polymorphonuclear leucocytes. × 500.

FIG. 6.—*Hodgkin's Disease, Grade* 3. Showing the loss of the normal architecture with a marked cellularity and many giant cells. × 100.

FIG. 7.—*Hodgkin's Disease, Grade* 3. Showing the pleomorphism of the cells with giant cells, conspicuous reticulo-endothelial overgrowth and mitotic activity. × 500.

2 3

4 5

6 7

*Hodgkin's disease, Grade* 3.

This grade of Hodgkin's disease has ill-defined limits ; at one end of the scale it merges into the Grade 2 type and at the other end with reticulum cell sarcoma. As Jackson and Parker (1947) state, the one diagnostic feature is the presence of a variable number of Dorothy Reed giant cells in an actively growing reticulo-endothelial cell proliferation. To add to the confusion, Jackson and Parker's photomicrographs are not diagnostically informative and Custer and Bernhard (1948) regard the Hodgkin's sarcoma as virtually synonymous with reticulum cell sarcoma.

However, there does appear to be a small number of cases in this series showing the characteristic cellular pleomorphism of Hodgkin's disease containing the diagnostic giant cells. In addition, there is an abnormally active reticulo-endothelial cell overgrowth with frequent mitotic figures and local areas of necrosis. The appearances resemble an actively growing tumour and the fibrous tissue, so prominent in the Grade 2 counterpart, is scanty. This type is called Hodgkin's disease, Grade 3, to denote a pathological process at the more cellular and malignant end of the scale of Hodgkin's disease (Fig. 6 and 7). In this sub-group the tempo of the disease is extremely rapid, and 12 of the 16 deaths occurred within 18 months of diagnosis. No case survived for more than 4½ years.

Other features are worthy of comment in this series as a whole. There is an apparent therapeutic success in female patients with Hodgkin's disease restricted to the mediastinum and cervical regions. This success may be due to confusion of the histological appearances with the granulomatous form of malignant thymoma (Eisenberg and Sahyoun, 1950 ; Thomson, 1955).

In 29 cases, serial biopsies were available. In 11 cases an increased cellularity of the Hodgkin's tissue was detectable in the subsequent pathological material as the disease progressed. We found no examples of complete cellular alteration necessitating a different diagnosis, as did Custer and Bernhard (1948).

### TREATMENT.

Bearing in mind the many factors influencing the course of Hodgkin's disease, it is difficult to assess the true value of any form of treatment. Of the 227 patients in this series, 14 were untreated ; 5 presented in Stage I, and 9 in Stage III. The average survival after diagnosis was under 4 months in the last group, and 41 months in the first. One Stage I case survived for just over 5 years. These cases cannot be considered an adequate control series. Firstly, the number was small and the overall figures must be unduly influenced by a few good or bad results. Secondly, there was definite selection of the Stage III cases, 4 being considered too advanced for any attempt at treatment. They do, however, emphasize again the totally different outlook in these two stages.

Surgical resection of the diseased area has been recommended as the ideal method of treatment, when technically possible (Baker and Mann, 1940). Slaughter and Craver (1942) quote 5 patients alive and well, 5, 6, 8, 11 and 11 years after removal of the affected lymph nodes by block dissection. Three of our patients were treated in this manner, but in each case the operation was followed by X-ray therapy. One patient had developed lymph glands in the adjacent supra-clavicular triangle by the time X-ray therapy was commenced, a few weeks after an apparently successful dissection. One case has survived for 5 years.

There does not appear to be much place for this method of treatment. If the disease is genuinely limited to one area, its extreme radio-sensitivity makes radical X-ray therapy the ideal method for general purposes. In very exceptional cases the disease may be so localised that complete resection is possible, and occasionally it may arise at some unusual site, such as the stomach, where adequate irradiation is difficult. Resection in this type of case would offer the only real hope of long survival (Slaughter and Craver, 1942).

Irradiation has been the main method of treatment in the majority of our cases. A few of the earlier patients were exposed to radium, either in the form of a surface applicator, teleradium, or needle implant. Since the earlier years all irradiation has been by means of 170–250 kv. X-rays, 1–3·8 mm. Cu h.v.l. Throughout various centres, there is considerable difference of opinion as to the most effective methods of treatment (Medinger and Craver, 1942 ; Peters, 1950 ; Levitt, 1952 ; Nice and Stenstrom, 1954). We have analysed all cases treated initially by deep X-rays, in an attempt to assess the dose necessary to ensure local cure of involved nodes. Excluding all Stage III cases, the remainder can be placed in three main groups. The first includes those cases with complete disappearance of the treated nodes, and no recurrence, locally or elsewhere, the patient known to have had no further trouble, for a period of 5 years or longer (Table VI). The

TABLE VI.—*Cases Without Recurrence after First Treatment.*

| Stage. | X-ray dosage (t.d.). | Survival (years). |
|---|---|---|
| I | 2000 r | 6½ |
| I | 2100 r | 5 |
| I | 3000 r | 8 |
| II | 3000 r | 12 |
| II | 3500 r | 19 |
| II | 4000 r | 14 |
| II | 3730 r | 8 |
| II | 6400 r | 11 |

t.d. : Tumour dose.

second includes all cases where disappearance of the nodes was followed by a first recurrence at the previously treated site (Table VII). By doing so, those cases are excluded where apparently local recurrence could have been caused by retrograde spread from adjacent involved areas. All cases in the second group received a dose of at least 2000 r and 6 received at least 3000 r. Amongst those cases with the first recurrence at the previously treated site, 13 were given 2200 r or less, and none received more than 3000 r. The total number of cases is small, but this suggests that complete local resolution is probable above a dose of 3000 r, while a local recurrence is more likely if less than this amount of X-ray therapy is given. From this it follows that reasonably localised disease should be treated to a level of at least 3500 r over 3½ to 4 weeks, provided that this is technically possible, if an attempt is to be made at a radical cure.

Wide field irradiation may be necessary in the later stages of the disease. Large volumes of body tissue cannot be raised to such dose levels with safety, and palliation of a more temporary nature is the most that can be expected.

The cytotoxic poisons have been used at the Middlesex Hospital with increasing frequency since their introduction by Wilkinson (Wilkinson and Fletcher, 1947).

TABLE VII.—*Cases with First Recurrence at Site of Previous Irradiation.*

| Stage. | X-ray dosage (t.d.). | Recurrence. |
|---|---|---|
| I | 400 r | 3 years |
| I | 1000 r | 4 ,, |
| I | 2000 r | 1 year |
| I | 3000 r | 1¼ years |
| I | 3000 r | 1 year |
| II | 1000 r | 2½ years |
| II | 1000 r | 5 ,, |
| II | 1050 r | 6 months |
| II | 1150 r | 4 ,, |
| II | 1500 r | 3 ,, |
| II | 1800 r | 6 ,, |
| II | 1800 r | 7 ,, |
| II | 2200 r | 6 ,, |
| II | 2200 r | 3 years |
| II | 2400 r | 10 months |
| II | 3000 r | 3 years |

t.d.: Tumour dose.

The majority of cases have been given nitrogen mustard, often in repeated courses. Their use has been well reviewed recently by Beatty and Howells (1954). There does not appear to be any evidence available that these agents are preferable to X-rays in the treatment of localised Hodgkin's disease. Almost all our cases treated with these agents had widespread disease, and it is therefore hardly surprising that responses have been short-lived. Nitrogen mustard was given as the first method of treatment in only 5 cases. Survivals after treatment were 34, 33, 23, 5 and 2 months. This is not a fair comparison with those cases treated by X-rays, as 4 of the 5 were in Stage III. The one early case, a girl of 23, with disease limited to the cervical glands on one side, had a remission of 9 months following which the disease progressed rapidly. The danger of severe and possibly fatal depression of all the bone marrow elements is common to both the cytotoxic poisons and to wide field irradiation. There does not appear to be any logical reason for their use in apparently localised disease, when local irradiation can be given to a high dose level without any severe general body effects. Even when indicated by widespread disease, care is needed in their administration, but provided adequate control is maintained they have an important place in the management of Hodgkin's disease.

### COMPLICATIONS.

Hodgkin's disease is almost invariably fatal. The exact cause of death may be difficult to ascertain; and was often so in this series. It was frequently difficult to obtain precise clinical details or post-mortem findings, as many of the patients died at home or in other institutions. In some cases the disease was so widespread at autopsy that the exact cause of death could not be decided. In spite of this, a review of the common complications occurring in this series revealed some points of importance. It is probable that in some cases their earlier recognition and more adequate treatment might have led to the prolongation of useful life.

*Pregnancy.*—As with tuberculosis, it has often been held that pregnancy is contra-indicated in Hodgkin's disease, in that it is frequently followed by an exacerbation of the disease process. Eight patients in this series became pregnant. We found no evidence that this occurrence altered an any way the tempo of the disease, but no reasonable conclusions can be drawn from such a small series. Stewart and Munro (1952) have reviewed the subject. After analysing 134 reported cases, they conclude that pregnancy has no predictable effect on the course of the disease.

*Blood abnormalities* occurred frequently as the disease progressed. An anaemia was the rule in the more advanced cases. This was not usually of a severe degree, and was correctable by transfusion and by treatment of the underlying disease. In some cases it became a major problem, and repeated transfusions failed to raise the haemoglobin above dangerously low levels. None of these cases had any obvious blood loss from any site. Two patients developed a haemolytic anaemia ; one as a terminal complication for which nothing could be done, while the other responded well to cortisone, followed by splenectomy.

Leucopenia and thrombocytopenia, often associated with a low haemoglobin level, were frequently found at a late stage in the disease. The majority of cases appear to have followed either invasion of the bone marrow or prolonged treatment with the cytotoxic poisons or X-rays. The leucopenia is often a deciding factor, preventing further treatment and predisposing the victim to secondary infections. It is therefore important to conserve the bone marrow for as long as possible, and it is probably advisable to avoid the use of cytotoxic poisons and wide field irradiation, unless they are indicated by generalised disease.

An eosinophilia is found in under 10 per cent of all cases of Hodgkin's disease. In our experience an increase of the eosinophil count above the normal range was found only when widespread disease was present, when there was macroscopic invasion of the skin, or when the patient complained of pruritus. We therefore consider that an eosinophilia, if present, suggests that the disease is widespread. The only exception to this was seen in those cases with skin invasion as the primary manifestation. As has been mentioned previously, the outlook in this type of case appears to be extremely variable, and an eosinophilia cannot then be considered a sign of poor prognosis.

*Intrathoracic spread* is extremely common, occurring in the majority of cases. Hilar gland enlargement is, of course, the commonest form, occurring at some stage in two-thirds of our cases. This finding does not appear to influence the prognosis adversely. Twenty-nine of the cases in Stage II when first seen had hilar gland enlargement on X-ray examination of the chest. Sixteen or 55 per cent, of these cases survived for more than 5 years Only one of the 227 patients developed clinical signs of superior vena caval obstruction.

The presence of parenchymal pulmonary involvement is, however, of more serious import. Diffuse infiltration of the lung fields was found commonly in the late stages of the disease, and 5 patients developed discrete rounded intrapulmonary opacities. No case with parenchymal infiltration responded satisfactorily to treatment. One patient died of haemorrhage from a main branch of the right pulmonary artery, which ruptured into the centre of a necrotic mass of Hodgkin's tissue.

*Intra-abdominal spread* involving the retroperitoneal glands occurs at some stage in the majority of patients with Hodgkin's disease.

In the present series, retroperitoneal gland involvement was found, either clinically, or at autopsy in over one half of all cases. Once this complication occurred the outlook became gloomy, as involvement of the renal tract and hepatic system were common sequelae. Probably a number of the cases of so-called " Hodgkin's cachexia " were unrecognised cases of uraemia. In addition the commonest site of extra-dural deposits of Hodgkin's tissue is in the lower dorsal and upper lumbar spine, presumably from direct spread backward from the retroperitoneal tissues. Thirteen of the 16 cases of paraplegia in this series occurred in this region.

The difficulties of treating a large mass of glands in the upper abdomen have already been mentioned, and diagnosis at an earlier stage may be extremely difficult. There may be nothing to feel on abdominal examination, but a history of recurrent attacks of upper abdominal pain, often radiating through to the middle of the back and waking the patient in the small hours of the morning is very suggestive of retroperitoneal spread. Intravenous pyelography may be a useful confirmatory investigation, demonstrating displacement of the kidneys or ureters. If no other cause can be found, irradiation of the affected area usually leads to a rapid improvement of symptoms, thus confirming the provisional diagnosis.

*Nervous system involvement* is a common complication of Hodgkin's disease.

In this series, 8 patients developed actual invasion of peripheral nerves, including the brachial plexus, cervical sympathetic chain, and oculomotor and recurrent laryngeal nerves. Two patients developed a multiple peripheral neuritis. All cases were advanced, and the ultimate prognosis was unaffected by these complications.

Coma was known to occur as a terminal event in at least 17 of the patients, associated in some cases with convulsions and preceded by mental disturbances. In some of these cases the clinical appearance of the patient suggested the possibility of the development of intra-cerebral deposits. In fact no such invasion was found in any case at post-mortem examination. This result is not unexpected, as there are only four acceptable examples of brain invasion in the world literature reviewed recently by Fein and Newill (1954). One patient was found at post-mortem examination to have an intra-cranial extradural deposit of the disease.

Extradural deposits with paraplegia developed in 16 cases, 9 without obvious change in the vertebral column on X-ray examination. The importance of the early recognition and treatment of this complication is well illustrated by one patient who lived a normal life for 4 years following laminectomy and irradiation of the affected cord segment, until further generalisation of the disease occurred. Paraplegia may develop at any state of the disease, and in 2 of the 16 cases it was the presenting symptom. This subject is well reviewed by Smith and Stenstrom (1948).

Herpes zoster occurred in 27 patients. This is a significant complication, as it was followed within 3 months by an exacerbation of the underlying Hodgkin's disease in all except 2 cases.

*Secondary infection.*—This complication is probably of less importance following the introduction of the antibiotics, but it is always potentially dangerous in the later stages of the disease, when the polymorphonuclear count is low.

Tuberculosis has long been associated with Hodgkin's disease, so much so that a common cause has often been postulated. Seven patients in this series developed tuberculous lesions. This does not appear to be excessively frequent in a disease which may be both debilitating and long-standing. Part, at any rate, of this traditional association may be due to misdiagnosis. Five case of Hodgkin's disease were thought, on clinical examination, to have tuberculous glands of the neck, and were treated as such for variable periods before the correct diagnosis was finally made by biopsy. One of these patients was considered tuberculous for over 4 years, one of which was spent in a sanatorium. In the late stages of the disease, pulmonary infiltration with Hodgkin's tissue is very common, giving X-ray appearances that may be indistinguishable from those seen in pulmonary tuberculosis.

In all our tuberculous patients the underlying Hodgkin's disease was already advanced and the outlook, therefore, extremely poor. Four deaths were undoubtedly directly due to tuberculosis. Presumably, with modern antibiotics, the prognosis is now better in this type of case.

*Complications unrelated to Hodgkin's disease.*

It is very easy to miss the development of a new disease in the course of a chronic illness. Additional complications may be attributed to the original complaint with fatal results. Three of our patients developed a perforation of a duodenal ulcer. Two were seen elsewhere, the pain was attributed to Hodgkin's disease and the correct diagnosis only established at post-mortem examination. One case was recognised and successfully dealt with. A proven peptic ulcer occurred in 7 patients, and was a constant source of difficulty in diagnosis, the pain being confused with that found with retro-peritoneal gland involvement. This high incidence may be attributed to the constant nervous tension under which the majority of these unfortunate patients must live.

### CONCLUSIONS.

The relative merits of the different forms of treatment in Hodgkin's disease cannot be evaluated successfully unless the cases under review are strictly comparable. An analysis of this series suggests that many factors affecting both the natural history and prognosis of the disease can be clearly recognised.

The important features which determine the eventual prognosis include the stage reached by the disease at the time of presentation, the histological appearances, the age at onset and the sex of the patient. In our experience the duration of the disease before treatment has proved of little prognostic value, as it is often impossible to assess this factor accurately.

Staging of the disease at the time of the initial clinical examination has proved of considerable value Of the patients placed in Stages I and II about 50 per cent are alive at 5 years, while those in Stage III at the time of presentation very rarely survive this period. During the earlier years covered by this series some of the patients included in Stages I and II were insufficiently investigated to exclude completely early generalisation of the disease. The ensuing short survival of many of these patients suggests that, with more searching clinical, radiological and haematological investigation, some of these cases would have been more accurately placed in the Stage III category of the disease. If this assumption is correct the outlook in the Stages I and II would compare even more favourably with Stage III.

There does appear to be some difference in the prognosis of the various age-groups and between the two sexes. An analysis of the results suggests that the best survival rate is found in young women in the third and fourth decades. Unfortunately, the number of patients in this series is small and further subdivision of the cases into groups according to the age and sex renders more detailed analysis of doubtful significance.

Histological examination of biopsy material is essential as the sole method of establishing the specific diagnosis. In the absence of histological proof both the diagnosis and, therefore, the prognosis remain entirely conjectural. It is possible to recognise three histological grades in Hodgkin's disease, each with a significantly differing average prognosis. All the patients with a Grade 1 microscopical appearance were alive 5 years after diagnosis, while no histologically Grade 3 patient survived this period. Taking each grade separately, it was not possible to decide on histological grounds alone which individual case would fare better or worse than the average within that grade.

Eighty-eight per cent of the cases of this series have a Grade 2 type of cellular picture, and there is no recognisable histological distinction between those patients who survive for 15 years and those who succumb to the disease in as many weeks. This is the major limitation of the histological grading of Hodgkin's disease. However, it is extremely valuable in assessing the prognosis as a whole to recognise both the Grade 1 and Grade 3 varieties of the disease, although these account for only a small percentage of the series.

Although many of the complications of Hodgkin's disease, such as parenchymal lung involvement and retroperitoneal spread, cannot be effectively treated, there remain a few where early recognition and treatment are of paramount importance. These include the development of paraplegia associated with extradural deposits of Hodgkin's tissue, acquired haemolytic anaemia, secondary infection including tuberculosis and the occurrence of peptic ulceration.

No dogmatic statement can be made as to the precise value of the treatment given to the patients in this series. The few untreated cases were largely selected in that the disease was advanced; similarly, the cytotoxic poisons were rarely used in either early cases or as the primary method of treatment. As regards irradiation, it does appear possible to eradicate local disease for a variable and, in some cases a considerable, length of time provided sufficient dosage is given. Heavy irradiation, in excess of 3000 r, is possible only when the disease is limited in extent, but it does appear a justifiable method of treatment in this type of case.

### SUMMARY.

Two hundred and twenty seven cases of Hodgkin's disease are reviewed. The prognosis in an individual case appears to depend on many factors, including the stage of the disease at the time of presentation, the age of onset, the sex of the patient and the histological appearance. Progress of Hodgkin's disease is modified by treatment, but the true value of any form of therapy cannot be assessed unless the other prognostic factors are considered.

We wish to thank Professor B. W. Windeyer, Miss M. D. Snelling, and the Medical and Surgical Staff of the Middlesex Hospital for permission to publish details of the cases under their care. We are grateful to Professor R. W. Scarff and Dr. A. C. Thackray for advice and encouragement at all stages of this investigation, and to the many pathologists who have so willingly provided us with histological material. We are indebted to Mr. T. Cowan and Miss Scott, of the Records Department, for their unsparing efforts, without which this work could not have been completed.

Part of the expense of this investigation was defrayed by the British Empire Cancer Campaign.

### REFERENCES.

BAKER, C. AND MANN, W. N.—(1940) *Lancet*, i, 23.
BEATTIE, J. W. AND HOWELLS, L. H.—(1954) *Quart. J. Med.*, New Series, **23**, 231.
CUSTER, R. P. AND BERNHARD, W. G.—(1948) *Amer. J. med. Sci.*, **216**, 625.
EISENBERG, S. J. AND SAHYOUN, P. F.—(1950) *Arch. Path.*, **49**, 404.
FEIN, S. B. AND NEWILL, V. A.—(1954) *Amer. J. Med.*, **17**, 291.
GILBERT, R.—(1939) *Amer. J. Roentgenol.* **41**, 198.
HARRISON, C. V.—(1952) *J. Path. Bact.*, **64**, 513.
JACKSON, H. AND PARKER, F.—(1947) 'Hodgkin's Disease and Allied Disorders.' New York (Oxford University Press).
LEVITT, W. M.—(1952) 'Handbook of Radiotherapy.' London (Harvey & Blythe).
LUMB, G.—(1954) 'Tumours of Lymphoid Tissue.' Edinburgh and London (E. & S. Livingstone Ltd.).
MEDINGER, F. G. AND CRAVER, L. F.—(1942) *Amer. J. Roentgenol*, **48**, 651.
NICE, C. M. AND STENSTROM, K. W.—(1954) *Radiology*, **62**, 641.
PETERS, M. V.—(1950) *Amer. J. Roentgenol.*, **63**, 299.
ROBB-SMITH, A. H. T.—(1947) 'Recent Advances in Clinical Pathology.' London (J. & A. Churchill Ltd.).
SLAUGHTER, D. P. AND CRAVER, L. F.—(1942) *Amer. J. Roentgenol.*, **47**, 596.
SMITH, M. J. AND STENSTROM, K. W.—(1948) *Radiology*, **51**, 77.
SMITHERS, D. W., RIGBY-JONES, P., GALTON, D. A. G. AND PAYNE, P. M.—(1952) *Brit. J. Radiol.*, Suppl. No. 4.
STEWART, H. L. AND MUNRO, R. W.—(1952) *Amer. J. Obstet. Gynaec.*, **63**, 570.
THOMSON, A. D.—(1955) *Brit. J. Cancer*, **9**, .
WILKINSON, J. F. AND FLETCHER, F.—(1947) *Lancet*, ii, 540.